# Book Review

**Published:** 1917-02

**Authors:** 


### BOOK REVIEW

### MANUAL OF NERVOUS DISEASES, BY IRWING J. SPEARS, M. D.—W. B. SAUNDERS CO. 1916.

To the average practitioner and student of medicine, the name Neurology is a bugaboo which frightens them away from a close enough scrutiny of this subject tomaster it even in part. The reason for this is a pre-conceived notion that it is impossible to have the anatomy and physiology of the Nervous System itself. Whereas, certain fundamental and rather elementary conceptions are all that are necessary to a good, square beginning.

Now, perhaps if such individuals could be gently and tenderly let within the domains of Neurology, having once found themselves safely within, they could and would make further —and to themselves rather surprising—progress.

We think this is time; and starting from this assumption, we find this little book of Dr. Spear a rather pleasing contrast to the many tomes which fill our Neurological library. Every part of it is admirable, from the anatomical and physiological sections on through to the end. We are of the opinion that the simplicity of the setting forth of the anatomy of the central nervous system will appeal to the average man and encourage him along this path which is strewn with hobgoblins. It is just such a conception as this which has always in our experience appealed to the average student of medicine.

The treatises on diseases per se are each brief, concise and well put· They are so presented that one gets a very clear conception of the disease in question.

As stated in the preface, Dr. Spear does not wish to make an exhaustive rendition of the subject matter, but wishes to present a book which will appeal to the average man and give him a running start into a preconceivedly difficult subject. As such a book we feel sure it will make a very strong appeal.

W. W. YOUNG.

